# Phenotypic and genotypic identification of streptococci and related bacteria isolated from bovine intramammary infections

**DOI:** 10.1186/1751-0147-55-53

**Published:** 2013-07-18

**Authors:** Andreas Raemy, Mireille Meylan, Simona Casati, Valeria Gaia, Beat Berchtold, Renate Boss, Anja Wyder, Hans U Graber

**Affiliations:** 1 Clinic for Ruminants, Department of Clinical Veterinary Medicine, Vetsuisse-Faculty, University of Berne, Bremgartenstrasse 109a, PO Box 8466, 3001 Berne, Switzerland; 2 Istituto Cantonale di Microbiologia, Via Mirasole 22a, 6500 Bellinzona, Switzerland; 3 Agroscope Liebefeld-Posieux Research Station ALP, Schwarzenburgstrasse 161, 3003 Berne, Switzerland

**Keywords:** Mastitis, Cattle, Streptococci, Identification, Mass spectroscopy

## Abstract

**Background:**

*Streptococcus* spp. and other Gram-positive, catalase-negative cocci (PNC) form a large group of microorganisms which can be found in the milk of cows with intramammary infection. The most frequently observed PNC mastitis pathogens (major pathogens) are *Streptococcus uberis*, *Strep. dysgalactiae*, and *Strep. agalactiae*. The remaining PNC include a few minor pathogens and a large nonpathogenic group. Improved methods are needed for the accurate identification and differentiation of PNC.

A total of 151 PNC were collected from cows with intramammary infection and conclusively identified by 16S rRNA sequencing as reference method. Nine phenotypic microbiological tests (alpha-hemolysis, CAMP reaction, esculin hydrolysis, growth on kanamycin esculin azide agar and on sodium chloride agar, inulin fermentation, hippurate hydrolysis, leucine aminopeptidase and pyrrolidonyl peptidase activity), multiplex PCR for the three major pathogens (target genes for *Strep. uberis*, *Strep. dysgalactiae* and *Strep. agalactiae*: *pauA*, 16S rRNA, and *sklA3*, respectively), and mass spectroscopy using the matrix-assisted laser desorption ionization-time of flight (MALDI-TOF MS) were evaluated for the diagnosis and discrimination of the three clinically most relevant PNC.

**Results:**

The probability that a strain of *Strep. uberis*, *Strep. dysgalactiae* and *Strep. agalactiae* was correctly identified by combining the results of the 9 phenotypic tests was 92%, 90%, and 100%, respectively. Applying the multiplex PCR, all strains of the three major pathogens were correctly identified and no false positive results occurred. Correct identification was observed for all strains of *Strep. uberis* and *Strep. agalactiae* using MALDI-TOF MS. In the case of *Strep. dysgalactiae*, some variability was observed at the subspecies level, but all strains were allocated to one single cluster.

**Conclusions:**

The results of the present study show that reliable identification of the clinically most relevant PNC (*Strep. uberis*, *Strep. agalactiae* and *Strep. dysgalactiae*) can be obtained by use of a combination of colony morphology, hemolysis type and catalase reaction, and a multiplex PCR with specific primers restricted to these 3 pathogens. The MALDI-TOF MS is a fast method that shows promising results, although identification of *Strep. dysgalactiae* at the subspecies level is not yet satisfactory.

## Background

Bovine mastitis is a worldwide problem in the dairy industry. It is a disease of major economic importance, causing reduced milk quality, loss in production and increased use of drugs and veterinary services worldwide [1]. One important group of bacteria associated with bovine intramammary infection (IMI) includes *Streptococcus (Strep.)* spp. and further Gram-positive, catalase-negative cocci, subsumed as PNC in the following [2,3]. The large group of PNC includes pathogenetic and apathogenetic bacteria. Pathogenic PNC are involved in clinical and subclinical mastitis and are typically observed in large dairy herds [4,5]. *Strep. uberis*, *Strep. dysgalactiae*, and *Strep. agalactiae* are prominent PNC pathogens involved in bovine mastitis. While, in the case of *Strep. uberis*, a high prevalence has been reported throughout the world [6,7], IMI caused by *Strep. agalactiae* has been rarely observed during the last decades in Switzerland, likely because it can be controlled by improved milking management and it shows good susceptibility to antibiotics [8]. A recent publication, however, suggests that *Strep. agalactiae* may regain clinical importance, particularly in herds milked by automatic milking systems [9]. *Strep. agalactiae* is an udder-associated pathogen, whereas *Strep. uberis* behaves mainly as an environmental pathogen [10], although Zadoks *et al*. [11] have shown that it can also be transmitted via the milking machine. *Strep. dysgalactiae* is mostly spread from cow to cow during milking, but cows may also become infected from environmental reservoirs [4,12]. Intramammary infections with the major PNC pathogens, *Strep. uberis*, *Strep. dysgalactiae*, and *Strep. agalactiae,* require antibiotic treatment whereas this is not necessary for apathogenic PNC [13]. Good antimicrobial susceptibility in vitro for penicillin is reported for these major pathogens [14,15]. However, practitioners often report unsatisfactory success rates for the treatment of mastitis caused by *Strep. uberis.* Lack of accurate bacteriological diagnosis and frustration about treatment results may lead to increased use of last resort antibiotics (personal communication by practitioners). As this aspect becomes more and more important and restricted use of antibiotics in the food producing industry is demanded by the consumers and legal authorities, fast and cost-effective analysis methods are needed for the identification of organisms causing IMI in order to allow for targeted treatment and optimized use of antibiotics in dairy cows [16,17].

Traditionally, mastitis pathogens have been identified by classical phenotypic microbiological procedures [4,10]. Valuable alternatives are given by methods based on PCR or on mass spectroscopy (MS). Various diagnostic improvements toward the identification of PNC have been made during the last years. A multiplex PCR method has been made commercially available, which appears to provide superior results as compared to common phenotypic tests and allows for identification of a broad spectrum of pathogens causing IMI within a few hours [18]. Lately, MS using the matrix-assisted laser desorption ionization-time of flight (MALDI-TOF) has emerged as a fast alternative for the identification of bacterial species. This method is based on analysis of the protein composition of bacteria resulting in mass spectra which may be considered as fingerprints of the cells. This method is considered to be reliable for identification of bacteria at the species level [19-21].

The present study was designed to evaluate and compare the performance of conventional bacteriology methods, of a multiplex PCR and of MALDI-TOF MS to diagnose *Strep. uberis*, *Strep. dysgalactiae*, and *Strep. agalactiae* and to differentiate them from other PNC isolated from milk samples originating from cows with IMI.

## Materials and methods

### Sample collection

A total of 151 PNC strains were included. Ninety-seven were randomly selected from isolates from the study of Moret-Stalder *et al.*[22]. These strains were isolated during a representative epidemiological study on the prevalence of *Staphylococcus aureus* in Switzerland. All cows were sampled twice in randomly selected farms. Besides the staphylococci described in that publication, PNC were isolated from these milk samples and investigated in detail in the study of Wyder *et al.*[23]. The remaining 54 strains (comprising *Strep. uberis* (n = 40), *Strep. dysgalactiae* subsp. *dysgalactiae* (n = 9), and *Strep. agalactiae* (n = 5)) were newly isolated from milk samples of cows with IMI in the frame of routine diagnostic milk cultures. A total of 58 *Strep. uberis*, 20 *Strep. dysgalactiae* and 9 *Strep. agalactiae*, as well as 64 other PNC were included in the study. All 151 strains had been identified prior to the present study by 16S rRNA sequencing combined with phylogenetic evaluation (reference method). All strains of the Viridans streptococci (VS) group in the present study and those used in Wyder *et al*. are identical and therefore not listed in detail. The Sanger sequencing procedure was performed by Microsynth (Switzerland) using two primers (16SUNI-L1 and 16SUNI-G1) as described by Wyder *et al.*[23,24].

### Microbiological analyses

The 151 strains under study were tested with 9 classical phenotypic tests [4,25]. The strains were plated onto Columbia agar plates containing 5% sheep blood (BA, Biomérieux Suisse S.A., Geneva, Switzerland) and incubated aerobically at 37°C. They were evaluated after 24 h and 48 h for type of hemolysis, morphology, catalase activity and Christie-Atkins-Munch-Petersen (CAMP) reaction as described in Wyder *et al.*[23]. Esculin hydrolysis (ESC) was tested using an agar containing 41 g/L of modified Edwards Medium (Oxoid, Basel, Switzerland) supplemented with 500 mg/L of ammonium iron (III) citrate (Sigma-Aldrich, Buchs, Switzerland) as an iron source and 18 g/L of agarose (Agar No. 3; Oxoid, Basel, Switzerland). Hundred-and-eleven isolates (including 25 *Strep. uberis* randomly selected from the original 58 and 13 *Strep. dysgalactiae* out of 20) were further assayed for growth on Kanamycin Esculin Azide agar (KM; Merck, Berne, Switzerland) and on sodium chloride agar (NACL) as described by Qadri *et al.*[26]. Hippurate hydrolysis (HIP) and inulin fermentation (INU) were also evaluated [25]. Furthermore, leucin aminopeptidase (LAP) and pyrrolidonyl peptidase (PYR) activity of the strains were assessed using the LAP Disk kit (Oxoid, Basel, Switzerland) and the BBL DrySlide PYR-Kit (BD, Franklin Lakes, NJ), respectively.

### PCR

A multiplex PCR was used for identification of *Strep. uberis*, *Strep. dysgalactiae* and *Strep. agalactiae*. For each of the 151 isolates, 1 to 4 colonies of a pure culture grown on BA for 18 h were resuspended in 100 μL of TEL-buffer containing 10 m*M* Tris/HCl and 10 m*M* EDTA, pH = 8.5. The samples were incubated at 95°C for 10 min and placed immediately on ice. The obtained lysates were then diluted 1:100 in H_2_O.

The multiplex PCR was performed using the following reaction mix (total volume = 25 μL): 1x HotStarTaq Master Mix (Qiagen, Hombrechtikon, Switzerland), 2.5 μL of diluted lysate, and 300 n*M* of each of the primers listed in Table 1. To detect *Strep. uberis*, *Strep. dysgalactiae*, and *Strep. agalactiae* by multiplex PCR, corresponding primers were designed using the Oligo 6 software (Molecular Biology Insights, Cascade, CO, USA). In the case of *Strep. uberis*, the *pauA* gene [GenBank: FJ196527] which codes for plasminogen activator A was selected. For *Strep. dysgalactiae*, the corresponding 16S rRNA gene was used [GenBank: AB002485], whereas the *sklA3* gene [GenBank: AM050626] coding for fibrinogen binding protein served as the target to detect *Strep. agalactiae*.

**Table 1:** Oligonucleotides used as primers for multiplex PCR in the present study

**Bacterium target gene (protein)**	**Primer**	**Sequence 5′ - 3′**	**Amplicon size (bp)**
*Streptococcus uberis*	GSub-S	TGA TTC CGA CTA CTA CGC TAG AT	723
*pauA* (plasminogen activator A)	GSub-AS	ATA CTT TGA GTT TCA CCG AGT TC	
*Streptococcus dysgalactiae*	GSdys-S	GTG CAA CTG CAT CAC TAT GAG	279
16S rRNA	GSdys-AS	CGT CAC ATG GTG GAT TTT C	
*Streptococcus agalactiae*	GSag-S	ATT GAT AAC GAC GGT GTT ACT GT	487
*sklA3* (fibrinogen binding protein)	GSag-AS	CAT AGT AGC GTT CTG TAA TGA TGT C	

The following cycling program was applied: 95°C for 15 min followed by 35 cycles including 94°C for 60 s, 58°C for 60 s and 72°C for 60 s. The PCR reaction was terminated by a final extension at 72°C for 10 min followed by cooling down to 4°C. The PCR products were analyzed by electrophoresis using a 1.3% agarose gel in TBE buffer (45 m*M* Tris-borate, 1 m*M* EDTA, pH = 8.3) containing GelRed (Biotium Inc., Hayward, CA) as described by the manufacturer. The stained gels were viewed using a standard UV transilluminator (312 nm).

### MALDI-TOF MS

Seventy-nine strains selected randomly out of the original 151 were grown on BA (Biomérieux Suisse S.A., Geneva, Switzerland) and incubated at 37°C in a 5% CO_2_ atmosphere for 24 h.

For each isolate, a single colony was spread in duplicate with a plastic loop onto wells of a 48-position stainless steel FLEXImass target plate (Shimadzu Biotech, Kyoto, Japan). The bacteria were overlaid with 1 μl of a matrix solution containing 30–40 mg of *α*-cyano-4-hydroxy-cinnamic acid (Sigma-Aldrich, Buchs, Switzerland) in a solution containing equal volumes of acetonitrile, ethanol, water and 3% trifluoroacetic acid (all from Sigma-Aldrich, Buchs, Switzerland).

MALDI-TOF MS analyses were performed in the positive linear mode in the range of 3’000 to 20’000 mass-to-charge ratio (m/z) with delayed positive ion extraction (delay time: 104 ns with a scale factor of 800) and an acceleration voltage of 20 kV on an Axima Confidence mass spectrometer (Shimadzu Biotech, Kyoto, Japan) equipped with a 50 Hz nitrogen laser (pulse width: 3 ns). For every spot, 50 profile spectra were averaged and used for further analysis. The resulting averaged spectrum of each strain was then internally calibrated using characteristic biomarker masses of streptococci.

For strain identification, the Samaris software (Anagnostec GmbH, Potsdam, Germany) was applied. It compares the calibrated mass spectrum to reference spectra of various strains with known identity (SuperSpectra) present in the corresponding database (SARAMIS v. 4.09; Anagnostec GmbH). In particular, similarity values were generated by recognizing consensus mass signals between the spectra of the known and the query strains, and by weighting the detected signals according to their specific information. Dendrograms were prepared using the simi-larity values and the single link agglomerative clustering algorithm (0.08% error) implemented in the Samaris software [27].

### Statistics

The results of the phenotypic analyses of the various strains were expressed as frequencies or probabilities of observing a positive result for each performed test in a given bacterium. In addition, an in-house computer program in C# (Microsoft, Redmond, WA) was developed to identify the bacteria using a series of binary tests. The output of the program is the probability that a strain exhibiting the test pattern belongs to the indicated species, an approach that is frequently used to identify bacteria by a set of different biochemical reactions as performed by various commercial tests (e.g. API 20 Strep, Biomérieux) [28]. Evaluation included the presence of alpha-hemolysis and the results of the CAMP reaction, growth on KM and NACL, as well as the outcome of the ESC, INU, HIP, LAP, and PYR tests. For each PNC species and test, the outcomes were then combined. If, under these conditions, >50% of the strains showed a positive or negative result, a value of “+” for positive or “-” for negative was attributed, respectively. These values were then considered as the typical outcome of a variable for a particular PNC species. For each species, a typical pattern was then obtained by joining the typical outcomes of each variable. The required probabilities were calculated based on the phenotypic analysis results.

The analytical sensitivity and specificity of the multiplex PCR were calculated separately for *Strep. uberis*, *Strep. dysgalactiae*, and *Strep. agalactiae*. In addition, the corresponding 95% confidence intervals (95% CI) and the values of *P* were computed using the “Binary” package of the R 2.7.2. software [29].

## Results

### Conventional phenotypic single tests

A high percentage of alpha-hemolysis was observed for strains of *Strep. dysgalactiae* (100%), for the group of VS (88%) [30], for *Aerococcus viridans (*87%), and for *Lactococcus garvieae* (67%). This percentage was low for *Strep. uberis* (3%) and *Enterococcus* spp. (1 out of 3 strains), and no hemolysis was observed for *Strep. agalactiae* and *Lactococcus lactis* subsp. *lactis* (Table 2).

**Table 2:** Evaluation of single phenotypic identification tests for Gram-positive, catalase-negative cocci

**Bacterium**	**n**	***α*H**^**1**^	**CAMP**^**1**^	**ESC**^**1**^	**KM**^**1**^	**NACL**^**1**^	**INU**^**1**^	**HIP**^**1**^	**LAP**^**1**^	**PYR**^**1**^
*Streptococcus uberis*	58	3%	9%	100%	8%^a^	0%^a^	100%^a^	92%^a^	100%^a^	68%^a^
*Streptococcus dysgalactiae*	20	100%	0%	0%	0%^b^	0%^b^	0%^b^	0%^b^	100%^b^	0%^b^
*Streptococcus agalactiae*	9	0%	100%	0%	0%	0%	0%	100%	100%	0%
Viridans streptococci	17	88%	0%	53%	47%	0%	24%	24%	88%	6%
*Enterococcus* spp	3	1/3	0/3	3/3	3/3	1/3	2/3	1/3	2/3	1/3
*Aerococcus viridans*	30	87%	0%	100%	3%	73%	13%	100%	3%	27%
*Lactococcus garvieae*	12	67%	0%	100%	0%	0%	17%	92%	100%	83%
*Lactococcus lactis*	2	0/2	0/2	2/2	1/2	0/2	2/2	2/2	2/2	1/2

All strains were isolated from milk of cows with intrammamary infection and previously identified by 16S rRNA sequencing. Data is expressed as percentage (%) of strains showing a positive reaction in the corresponding phenotypic tests.

^1^Alpha-hemolysis (*α*H); CAMP reaction (CAMP); esculin hydrolysis (ESC); growth on kanamycin esculin azide agar (KM); growth on sodium chloride agar (NACL); inulin fermentation (INU); hippurate hydrolysis (HIP); leucine aminopeptidase activity (LAP); pyrrolidonyl peptidase activity (PYR).

^a^For these analyses, 25 strains of *Streptococcus uberis* were used.

^b^For these analyses, 13 strains of *Streptococcus dysgalactiae* were used.

All strains of *Strep. agalactiae* were CAMP-positive (100%). All other bacterial strains tested negative except for 5 strains (9%) of *Strep. uberis*.

All strains of *Strep. uberis*, *Enterococcus* spp., *A. viridans*, *L. garvieae*, and *L. lactis* showed a positive ESC reaction. In the case of VS, 53% of the isolates gave a positive result, whereas all strains of *Strep. dysgalactiae* and *Strep. agalactiae* tested negative.

The detailed results of all phenotypic tests are given in Table 2.

### Combination of the results of conventional phenotypic tests

Based on the simultaneous use of all binary phenotypic variables (Table 3), the probability that a strain of *Strep. uberis* exhibiting the typical test pattern actually belonged to this taxon was 92%. Better probabilities were achieved for *Strep. uberis* strains with divergent patterns (CAMP positive or PYR negative, 99% and 96%, respectively).

**Table 3:** Combination of phenotypic tests for identification of Gram-positive, catalase-negative cocci

**Bacteria**	**Test pattern**^**1**^	**Strain characteristics**	**Probability**	**Number/pattern**
*Streptococcus uberis*	– – + − − + + + +	Typical	92%	14/25
	– + + − − + + + +	CAMP-positive	99%	1/25
	– – + − − + + + −	PYR-negative	96%	7/25
*Streptococcus dysgalactiae*	+ − − – – – – + −	Typical	90%	13/13
*Streptococcus agalactiae*	– + − − – – + + −	Typical	100%	9/9
Viridans streptococci	+ − + − − – – + −	Typical	88%	2/17
	+ − − – – – – + −	ESC-negative	10%	2/17
	+ − + + − − – + −	KM-positive	100%	1/17
*Aerococcus viridans*	+ − + − + − + − −	Typical	100%	11/30
	+ − + − − – + − −	NACL-negative	96%	5/30
	+ − + − + − + − +	PYR-positive	100%	4/30
	– – + − + − + − −	*α*H-negative	100%	2/30
*Lactococcus garvieae*	+ − + − − – + + +	Typical	99%	4/12
	– – + − − – + + +	*α*H-negative	97%	3/12
	+ − + − − – + + −	PYR-negative	66%	2/12

All strains were isolated from milk of cows with intramammary infection. For each species and test, the outcomes were merged. If >50% of the strains showed a positive or negative result, “+” for positive or “-” for negative was attributed, respectively. Each value was then considered as the typical outcome of a variable for a particular bacterial species. For each species, a typical pattern was obtained by joining the typical outcomes of each variable. The probability was then calculated that a strain exhibiting the pattern belongs to the indicated species.

^1^The included tests are indicated in the following order: alpha-hemolysis; CAMP reaction; esculin hydrolysis; growth on kanamycin esculin azide agar; growth on sodium chloride agar; inulin fermentation; hippurate hydrolysis; leucine aminopeptidase activity; pyrrolidonyl peptidase activity.

Only one pattern was observed both for *Strep. dysgalactiae* and for *Strep. agalactiae*. According to these patterns, the probabilities of correct identification for *Strep. dysgalactiae* and *Strep. agalactiae* were 90% and 100%, respectively. The probabilities of other PNC were also calculated and are listed in Table 3.

### Multiplex PCR

**Figure 1:**
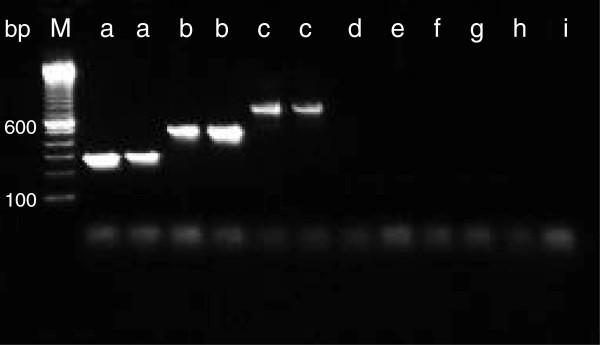
**Gel electrophoresis of PNC.** Gel electrophoresis of PCR products amplified by the multiplex PCR specific for *Streptococcus uberis*, *Streptococcus dysgalactiae*, and *Streptococcus agalactiae*. The amplicons were analyzed by electrophoresis using a 1.3% agarose gel and GelRed stain. Marker (M), 100-bp DNA ladder; *Streptococcus dysgalactiae* (**a**); *Streptococcus agalactiae* (**b**); *Streptococcus uberis* (**c**); *Streptococcus parasanguinis* (**d**); *Streptococcus oralis* (**e**); *Enterococcus faecalis* (**f**); *Aerococcus viridans* (**g**); *Lactococcus garvieae* (**h**); no template control (**i**).

The analytical sensitivity of PCR for *Strep. uberis* was 100% (*P <* 0.001; 95% CI: 94% to 100%). The same value was obtained for the analytical specificity (*P <* 0.001; 95% CI: 96% to 100%). The analytical sensitivity for *Strep. dysgalactiae* was 100% (*P <* 0.001; 95% CI: 83% to 100%), as well as the analytical specificity (*P <* 0.001; 95% CI: 97% to 100%). All strains of *Strep. agalactiae* (n = 9) were identified correctly by PCR; the analytical specificity was 100% (*P <* 0.001; 95% CI: 97% to 100%) (Figure 1).

### MALDI-TOF MS

**Figure 2:**
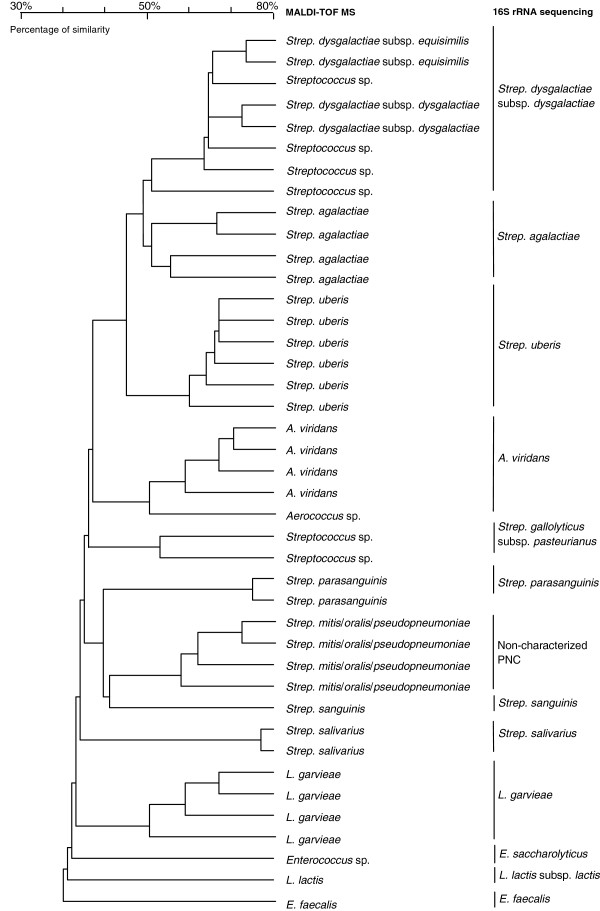
**Dendrogram for comparison of identification by MALDI-TOF MS with 16S rRNA sequencing.** Identification of Gram-positive, catalase-negative cocci (PNC) isolated from milk samples of cows with intramammary infection by sequencing the 16S rRNA gene (reference method) and by mass spectroscopy (MS) using the matrix-assisted laser desorption ionization-time of flight (MALDI-TOF) technology. An illustrative subset of 41 strains is depicted in the dendrogram. The degree of similarity is illustrated in percentage by the scale. Identification included *Streptococcus uberis* (*Strep. uberis*), *Streptococcus dysgalactiae* subsp. *dysgalactiae* (*Strep. dysgalactiae*), *Streptococcus agalactiae* (*Strep. agalactiae*) and other streptococcal species, *Aerococcus viridans* (*A. viridans*), *Enterococcus faecalis* (*E. faecalis*), *Enterococcus saccharolyticus* (*E. saccharolyticus*), *Lactococcus garvieae (L. garvieae*), and *Lactococcus lactis* (*L. lactis*). *Strep. mitis*/*oralis*/*pseudopneumoniae*: the indicated taxon may be a member of *Strep. mitis*, of *Strep oralis*, or of *Strep. pseudopneumoniae.*

The 79 strains used for evaluation of the MALDI-TOF MS method produced useful spectra to identify PNC at the species or subspecies level. All isolates belonging to the same species clustered consistently in the same group (Figure 2). In particular, the analysis revealed similar clusters for *Strep. uberis*, *Strep. dysgalactiae*, and *Strep. agalactiae*. These clusters were also clearly separated from those of other streptococci, *Enterococcus* spp., *A. viridans*, *L. garvieae*, and *L. lactis*. Correct identification was observed for all *Strep. uberis*, *Strep. agalactiae*, *Strep. salivarius*, *Strep. sanguinis*, *Enterococcus faecalis*, *L. garvieae*, and *L. lactis*. In the case of *Strep. dysgalactiae*, 2 strains were correctly identified at the subspecies level as *Strep. dysgalactiae* subsp. *dysgalactiae*, whereas 3 were identified as *Strep. dysgalactiae* subsp. *equisimilis* and 4 as *Streptococcus* spp. All *Strep. dysgalactiae* strains belonged to the same cluster. *Enterococcus faecalis* was correctly identified by MALDI-TOF MS whereas the 2 strains of *Enterococcus saccharolyticus* were only identified as *Enterococcus* spp.. Enterococcal species were located in 2 distant clusters in the dendrogram (Figure 2).

## Discussion

The PNC present in aseptically collected milk samples of cattle with IMI comprise a large group of *Streptococcus* spp., *Lactococcus* spp., *Enterococcus* spp., and *Aerococcus* spp. [23]. In spite of this large spectrum, *Strep. uberis*, *Strep. dysgalactiae*, and *Strep. agalactiae* are important mastitis pathogens whereas the remaining PNC are less frequently observed or nonpathogenic. Thus, it appears reasonable, at least for the situation observed in Swiss farms, to focus the diagnostics on these 3 major PNC pathogens [8].

Traditionally, bacterial identification has been performed by conventional phenotypic methods [4,10]. The results of the present study indicate that the power of these methods to discriminate PNC is limited. In the case of *Strep. uberis,* the combination of the results of 9 classical tests resulted in a probability of 92% that a strain showing the pattern defined as typical for this organism was actually a member of this taxon. These findings are in good agreement with the results of Odierno *et al.*[31] who achieved correct identification in 94% of the cases by use of 11 biochemical tests. Khan *et al.*[32] described the cultural, biochemical, serological and molecular properties of 132 strains of *Strep. uberis*. While all *Strep. uberis* strains in the present study were also ESC positive, which is in good agreement with the findings of the cited publications, other PNC (i.e. *L. garvieae* and *A. viridans*) were 100% ESC positive as well. These results confirm that correct phenotypic differentiation of PNC requires the use of multiple tests. The present study also demonstrates the phenotypic variability within the *Strep. uberis* species as the present strains differed from those of Khan *et al.*[31] with respect to the frequency of alpha-hemolysis and of positive reactions for INU, HIP and PYR. The probability of correct identification was lower for *Strep. dysgalactiae* (90%) than for *Strep. uberis*, but high for *Strep. agalactiae* (100%). Performing 9 phenotypic tests requires considerable material and manpower so that only a few of them are carried out routinely by diagnostic laboratories. In particular, final identification of *Strep. agalactiae* is usually based on the CAMP reaction alone. Indeed, all *Strep. agalactiae* strains of the present study showed a positive reaction. Only a few strains of *Strep. uberis* also showed positive results. However, using the probability calculation of Willcox (1973) [28] for the CAMP reaction alone, the probability was only 87% that an unknown PNC with a positive reaction actually belonged to the *Strep. agalactiae* taxon. Similar results have been described previously [4]. Nevertheless, the CAMP reaction as a single test seems to possess the best discriminatory power in the present study.

Some strains of *Strep. uberis* and *L. garvieae* showed very similar phenotypic properties. These findings indicate that discrimination of these bacteria is difficult if only one or a few of the evaluated phenotypic assays including ESC are used, therefore false identification may happen. As a consequence, the occurrence and importance of *L. garvieae* as a mastitis pathogen might have been underestimated in the past.

The multiplex PCR used in this study showed a high analytical sensitivity and specificity. All strains of the 3 major pathogens were correctly identified and no false positive results were noticed, which is in good agreement with the results of Phuektes *et al.* (2001) [7] or Gillespie *et al.* (2005) [33]. Koskinen *et al.* (2010) showed similar results for a commercially available multiplex PCR (Patho Proof®) [18]. Unfortunately, as some primers applied in this particular commercial PCR test are not published, the analysis of milk samples under monopoly conditions is considerably more expensive than the classical culture method. Phenotypic methods are therefore favored by many practitioners. The results of the present study show that reliable identification of the clinically most relevant PNC (*Strep. uberis*, *Strep. agalactiae* and *Strep. dysgalactiae*) can be obtained by use of a combination of colony morphology, hemolysis type, and catalase reaction as a first identification for triage and a multiplex PCR with specific primers restricted to these 3 pathogens.

The newer method MALDI-TOF MS appears to be a reliable tool to identify the entire spectrum of relevant bovine PNC associated with IMI. Indeed, a high level of agreement between this method and 16S rRNA sequencing (used here as the reference method for all isolates in the study) was observed for most of the PNC including *Strep. uberis*, *Strep. agalactiae*, and *L. garvieae*. Unsatisfying results, however, were observed for *Strep. dysgalactiae*. In comparison with 16S rRNA sequencing, only 2 analyzed strains were identified as *Strep. dysgalactiae* subsp. *dysgalactiae*. These findings are in agreement with those of Bizzini *et al.*[34] and Van Veen *et al.*[35] who suspected that the discordant results were due either to difficulties to differentiate among closely related organisms, such as e.g. *Strep. dysgalactiae* and *Streptococcus pyogenes*, or to insufficient reference spectra in the MALDI-TOF MS database. In the present study, all analyzed strains of *Strep. dysgalactiae* were allocated to the same cluster by the MALDI-TOF MS method. Although there were difficulties at the subspecies level, identification by MALDI-TOF MS was always correct at the species level when the broad cluster variation observed in the present study was considered. Correct identification at least at the species level, as achieved in the present study, should thus be sufficient for clinical purposes. The MALDI-TOF MS is fast and allows the identification of a broad spectrum of bacteria aside of PNC. These properties support the use of this methodology in diagnostic laboratories [19]. The authors determine a major drawback of MALDI-TOF MS, at least at present, in the fact that the necessary devices are very expensive, which limits their use in routine diagnostic. In contrast, PCR is established for routine milk analyses in diagnostic laboratories. However, compared to MALDI-TOF MS, the spectrum of bacteria covered by one analysis is limited as it depends on the number of primer pairs included in one PCR. The spectrum can be enlarged by running several PCR in parallel (i.e. multiplex PCR). Using milk culturing on BA, the combination of colony morphology, hemolysis type, and catalase reaction allows a first identification for triage. Based on these easy and fast steps, final identification of *Strep. uberis*, *Strep. dysgalactiae*, and *Strep. agalactiae* by the presented PCR approach is efficient and highly specific. Indeed, all strains of these 3 pathogens were correctly identified and differentiated from the other PNC. Final results can be obtained within 12 hours.

Despite the low numbers of some strains, the results of the present study suggest that the combination of limited conventional microbiology and PCR with a fast DNA extraction protocol allow rapid identification of major mastitis pathogens belonging to the PNC group. This approach is expected, therefore, to be affordable for routine diagnostic laboratories, with the benefit of a considerably more accurate identification of PNC than can be achieved using even a combination of multiple conventional tests.

## Conclusions

The results of the present study indicate that MALDI-TOF MS and PCR are both efficient methods to safely identify bovine IMI-associated PNC at the species level. MALDI-TOF MS is a fast method showing promising results and might supplant the traditional phenotypic methods in the future as these tests alone or in combination are less powerful and particularly not satisfying for the identification of *Strep. uberis* and *Strep. dysgalactiae*, two of the most important PNC mastitis pathogens. Reliable identification of the clinically most relevant PNC (*Strep. uberis*, *Strep. agalactiae* and *Strep. dysgalactiae*), and thus differentiation from other PNC, can be obtained by use of a combination of limited conventional microbiology (colony morphology, hemolysis type, and catalase reaction) as a first identification for triage and a multiplex PCR with specific primers restricted to these 3 pathogens involved in bovine mastitis.

## Abbreviations

IMI: intramammary infection; Strep. spp: *Streptococcus* spp; PNC: Gram-positive, catalase-negative cocci; MS: mass spectroscopy; MALDI-TOF: matrix-assisted laser desorption ionization-time of flight; BA: blood agar; CAMP: Christie-Atkins-Munch-Petersen reaction; ESC: Esculin hydrolysis; KM: Kanamycin Esculin agar; NACL: Sodium chloride agar; HIP: Hippurate hydrolysis; INU: Inulin fermentation; CI: Confidence interval; VS: Viridans streptococci; A. viridans: *Aerococcus viridans*; L. garvieae: *Lactococcus garvieae*; L. lactis: *Lactococcus lactis* subp. *lactis*.

## Competing interests

The authors declare that they have no competing interests.

## Authors’ contributions

AR and HUG designed and coordinated the study; AR was the main investigator and the principal author of the manuscript, under the supervision of HUG; MM participated in the study design and coordination, and in the preparation and redaction of the manuscript; RB, AW and BB collected milk samples and carried out the microbiological and molecular analyses; SC and VG carried out the mass spectrometry (MALDI-TOFF) analyses. All authors have read and approved the final manuscript.
